# Microbiological safety and antimicrobial resistance profiles of ready-to-eat seafood in Bayelsa, Nigeria

**DOI:** 10.1093/sumbio/qvae017

**Published:** 2024-07-13

**Authors:** Faith I Omeje, Abeni Beshiru, Isoken H Igbinosa, Etinosa O Igbinosa

**Affiliations:** Applied Microbial Processes & Environmental Health Research Group, Faculty of Life Sciences, University of Benin, PMB 1154, Benin City, 300283, Nigeria; Department of Microbiology, Federal University Otuoke, PMB 126, Yenagoa, 561502 Bayelsa, Nigeria; Applied Microbial Processes & Environmental Health Research Group, Faculty of Life Sciences, University of Benin, PMB 1154, Benin City, 300283, Nigeria; Department of Microbiology, College of Natural and Applied Sciences, Western Delta University, PMB 10, Oghara, 300104, Nigeria; Applied Microbial Processes & Environmental Health Research Group, Faculty of Life Sciences, University of Benin, PMB 1154, Benin City, 300283, Nigeria; Department of Environmental Management and Toxicology, Faculty of Life Sciences, University of Benin, PMB 1154, Benin City, 300283, Nigeria; Applied Microbial Processes & Environmental Health Research Group, Faculty of Life Sciences, University of Benin, PMB 1154, Benin City, 300283, Nigeria

**Keywords:** seafood, *Escherichia coli*, antimicrobial resistance, food contamination, biofilm, Nigeria, public health

## Abstract

The global significance of processed seafood necessitates vigilant monitoring for health risks, particularly in the ready-to-eat (RTE) sector. This study assessed the microbiological safety and antimicrobial resistance (AMR) profiles of 520 RTE seafood samples collected from Bayelsa, Nigeria. *Escherichia coli* identification was conducted using culture-based and polymerase chain reaction (PCR) methods. The isolates were further characterized using standard bacteriological techniques. PCR screening was employed to detect virulence and resistance genes. Of the processed RTE seafood samples assessed, 12.1% tested positive for *E. coli*. Extended-spectrum beta-lactamase (ESBL)-producing *E. coli* accounted for 2.3% of the samples. Various diarrheagenic determinants were identified, with aggregative adherence regulator-activated island (*aaiC*) and attaching and effacing (*eae*) being the most prevalent. Higher AMR levels were observed in ESBL-producing strains. Additionally, extracellular virulence factors, biofilm formation, and hydrophobicity assays revealed diverse pathogenic potentials among the isolates. The detection of beta-lactamase AMR genes, such as *bla_TEM_* (15.9%), *bla_CTX−M−15_* (3.2%), and *bla_CTX−M−1_* (1.6%), underscores the genetic mechanisms responsible for resistance in *E. coli* strains recovered from RTE seafood. These findings underscore the need for thorough monitoring and strict control procedures to ensure the safety of RTE seafood and mitigate the risks associated with AMR in food consumption.

**Sustainability Statement**

This research makes a substantial contribution toward Goal 2: Zero Hunger—ending hunger, improving nutrition, promoting sustainable agriculture, and ensuring food security. Through the identification and evaluation of *Escherichia coli* prevalence and its antimicrobial resistance patterns in ready-to-eat seafood, the study advances food safety and security efforts. Ensuring the microbiological safety of seafood products is crucial for preventing foodborne illnesses and improving nutrition. Additionally, promoting sustainable agriculture involves safeguarding food production systems from contamination, which this study addresses by highlighting potential risks associated with seafood consumption. Ultimately, the study contributes to achieving Goal 2 by supporting efforts to end hunger and ensure sustainable food systems by enhancing the safety and security of seafood products.

## Introduction

Processed seafood plays a crucial role in food systems globally, providing sustenance and livelihoods worldwide (Ramires et al. 2020). However, ensuring the safety and quality of processed seafood, particularly in the ready-to-eat (RTE) sector, presents a significant challenge. RTE seafood items are particularly vulnerable to contamination by diverse pathogens, such as *Escherichia coli*, which can pose notable health hazards to consumers if not adequately addressed. *Escherichia coli*, a recognized indicator of fecal contamination, is linked to various foodborne illnesses, including diarrhea, urinary tract infections, and severe conditions like hemolytic uremic syndrome (Sabala et al. 2021). Although most *E. coli* strains are harmless, some pathotypes have evolved genetic traits, enabling them to cause widespread illness and adapt to various environments (Sivakumar et al. 2021). These pathogenic variants, alternatively termed pathovars or pathotypes, are primarily classified according to molecular markers linked to particular disease manifestations. Virulent *E. coli* strains trigger diverse diseases through various mechanisms, showing significant genetic variability within and between pathotypes (Sánchez et al. 2021). The presence of *E. coli* in seafood often indicates unsanitary conditions and may endanger consumers, particularly when they are pathogenic, such as diarrheagenic *E. coli* (DEC) (Mohamed et al. 2009). Contamination of seafood with pathogenic *E. coli* strains raises serious public health concerns, as it can result in widespread outbreaks and significant morbidity and mortality rates.

Among DEC, which constitutes the primary subgroup implicated in gastrointestinal infections resulting from the consumption of contaminated foods, various pathotypes have been recognized. These include enteropathogenic (EPEC), enterotoxigenic (ETEC), enteroaggregative (EAEC), Shiga toxin-producing (STEC), diffusely adherent (DAEC), and enteroinvasive (EIEC) strains (Nguyen et al. 2005). Every *E. coli* pathotype possesses distinct genetic elements encoding virulence factors that influence host biology and disease progression. Although *E. coli* can be susceptible to heat treatments during seafood processing or cooking, the potential for cross-contamination persists, especially during post-processing tasks, thereby facilitating the survival of the pathogen (EFSA Panel on Biological Hazards et al. 2024). Consequently, seafood could become a primary avenue for the spread of disease-causing *E. coli*.

Various factors, including inadequate handling, processing, and sanitation along the seafood supply chain, impact the occurrence of *E. coli* in seafood products. These factors contribute to the contamination of seafood with pathogenic strains of *E. coli*, posing a substantial public health issue. In RTE seafood, the presence of *E. coli* is alarming due to its potential to cause illness and propagate antimicrobial resistance (AMR). Understanding the extracellular virulence factors, biofilm formation profiles, and hydrophobicity characteristics of *E. coli* isolates from such sources is essential for assessing their pathogenic potential and persistence in seafood products. The emergence of strains of *E. coli* producing extended-spectrum β-lactamase (ESBL) adds complexity to public health concerns (Beshiru et al. 2023), emphasizing the urgent need for effective surveillance and control measures.

Antimicrobial resistance *E. coli* strains pose a serious challenge as they can make traditional treatments ineffective, leading to more severe infections and higher healthcare expenses. The incidence of multidrug-resistant (MDR) bacteria continues to increase, with factors such as exposure to antimicrobial drugs contributing to this trend. The World Health Organization (WHO) has acknowledged AMR as a key public health issue, cautioning that diseases once thought eradicated could resurface as significant global threats because of the widespread existence of antimicrobial resistance genes (ARGs) and resistant pathogens (WHO 2023). Available data suggest a high incidence of AMR bacteria in seafood and related products in developing nations, likely stemming from the inappropriate or unregulated use of antimicrobials in aquaculture practices. Therefore, investigating AMR in developing countries, notably Nigeria, holds paramount importance.

In regions like Bayelsa, Nigeria, where seafood consumption is prevalent, understanding the microbiological safety and AMR profiles of RTE seafood products is crucial for safeguarding public health and promoting food security. Bayelsa’s geographical location along the coastline makes it a hub for seafood trade, highlighting the importance of comprehensive surveillance and monitoring programs to prevent and control foodborne illnesses. Therefore, this study aims to address these gaps by conducting a comprehensive assessment of RTE seafood products collected from shops and open markets in Bayelsa, Nigeria. This study seeks to provide valuable insights into the microbiological safety and AMR patterns of RTE seafood in the region by focusing on the identification of *E. coli* strains, determination of their AMR profiles, characterization of extracellular virulence enzymes, biofilm formation, and adherence capabilities, as well as screening for resistance and virulence genes. Additionally, identifying AMR patterns among *E. coli* isolates can support antimicrobial stewardship initiatives and promote judicious antimicrobial use in clinical and agricultural settings.

## Materials and methods

### Description of the study site and sample collection

The study’s sample size was determined using a formula:


\begin{eqnarray*}
{\mathrm{Sample}}\left( {{N}} \right) = \frac{{{{\left( {{{Z1}} - \propto /2} \right)}}^2{{P}}\left( {1 - {{P}}} \right)}}{{{{{d}}}^2}}.
\end{eqnarray*}

The formula considers the standard normal variant at 5% type I error rate (*Z_1−α/2_*), expected prevalence (*P*) from previous studies (ranging from 17.5% to 75.6%) (Rocha et al. 2014; Akande and Onyedibe 2019), and a set precision of 5% (*d*). Initially, the formula suggested a maximum sample size of 380. However, a total of 520 RTE seafood samples were procured from major open markets in Bayelsa, Nigeria, spanning April 2021 to January 2023. Specifically, Kpansia, Opolo, and Swali open markets were chosen due to their accessibility, strategic locations, and high patronage. Samples were procured based on the type of RTE seafood available (smoked fish, dried fish, fish sauce, crayfish, prawns and shrimps, seafood soups and stews, seafood snacks, and canned seafood) with respect to the sampling location. The availability of specific seafood products may vary depending on the local fishing industry and regional preferences. Seafood exerts a notable influence on the culinary traditions of Bayelsa State, and the processing methods may vary based on local customs and practices. The purchased RTE seafood was conveyed in a sterile polythene bag, placed on ice, and conveyed to the laboratory for microbiological analyses within 24 h after collection.

### Isolation of *E. coli*

Twenty-five grams of RTE seafood samples underwent homogenization in sterile 225 ml Tryptone Soy Broth (TSB) (Lab M, Lancashire, UK), leading to an initial dilution (10^−1^). The homogenization lasted for 1 min at 800 rpm using a shaker, followed by serial dilutions ranging from 10^−1^ to 10^−5^. A volume of 100 µl of each dilution was plated onto three Chromocult Coliform Agar plates (Merck Darmstadt, Germany), ensuring even distribution across the agar surface. Plates were maintained in an upright position until the inoculum was absorbed, typically taking around 10 min on adequately dried plates. Subsequently, the plates were incubated at 37°C for 24 h. Colonies exhibiting a purple/violet color, suggestive of presumptive *E. coli*, were counted and measured in colony-forming units per gram (CFU/g). After detecting purple/violet colonies on Chromocult Coliform Agar, isolated colonies were additionally cultivated on MacConkey agar (Lab M, Lancashire, UK), then incubated at 37°C for 24 h. Standard *E. coli* colonies, exhibiting dry, flat, non-mucoid, pink characteristics on MacConkey agar, were aseptically isolated and streaked onto nutrient agar medium (Lab M, Lancashire, UK) for purification, followed by an incubation period of 24 h at 37°C. The purity of the *E. coli* samples was validated using Analytical Profile Index (API) 20E tests, following the manufacturer's guidelines, and analyzed utilizing the API database (version 4.1) and APIweb™ identification software. Following purification and confirmation, *E. coli* specimens were preserved on nutrient agar slants at 4°C until needed for subsequent research purposes. *Escherichia coli* ATCC 25922 served as a control strain.

### DNA extraction and polymerase chain reaction amplification *E. coli*

Isolated and purified samples, subjected to DNA extraction, were revived by placing them in TSB and incubating them at 37°C for 18 h. DNA extraction was done utilizing the PeqGold Bacterial DNA kit (Peqlab Biotechnologie GmbH, Germany) based on the manufacturer’s instructions. Quality and quantity of total genomic DNA were assessed using a NanoDrop TM 1000 Spectrophotometer (Thermo Fischer Scientific, USA) and agarose gel electrophoresis. The *uidA* gene was amplified using the *E. coli*-specific primer pair (Table 1). *Escherichia coli* ATCC 25922 served as a control strain. Polymerase chain reaction (PCR) reactions (50 μl) included 10 μl gDNA, 2.5 μl R primer (adjusted to 10 pmol/μl), 2.5 μl F primer (adjusted to 10 pmol/μl), 5 μl PCR buffer with MgCl_2_, 23.7 μl nuclease-free water and 0.3 μl Taq polymerase, and 6 μl dNTP mix (Bej et al. 1991). PCR was carried out by employing the Peltier-Based Thermal Cycler (Zhengzhou Mingyi Instrument, China). Gel electrophoresis employed a 1.0% agarose gel with GelRed, and the gel ran for one hour at 100 V DC voltage.

**Table 1.** Oligonucleotide primer sequences for amplification of *E. coli* isolates, virulence-associated, and ARGs.

Target genes	Primer sequences (5′–3′)	Amplicon size (bp)	Annealing temperature	References
*Escherichia coli* (*uidA*)	uidA-F-AAAACGGCAAGAAAAAGCAG uidA-R-ACGCGTGGTTAACAGTCTTGCG	147	58°C	Bej et al. (1991)
AggR-activated island C (*aaiC*)	AAIC-F-ATTGTCCTCAGG CATTTCAC AAIC-R-CGACACCCCTGATAAACAA	215	57°C	Talukdar et al. (2013)
Anti-aggregation protein transporter (*aat*)	Pcvd432-F-CTGGCGAAAGACTGTATCAT pcvd432-R-CAATGTATAGAAATCCGCTGTT	650	57°C	Mohamed et al. (2009)
Attaching and effacing (*eae*)	eae-F-CCCGAATTCGGCACAAGCATAAGC eae-R-CCCGGATCCGTCTCGCCAGTATTCG	881	57°C	Oswald et al. (2000)
Bundle-forming pilus (*bfp*)	bfpA-F-GGAAGTCAAATTCATGGGGG bfpA-R-GGAATCAGACGCAGACTGGT	300	57°C	Talukdar et al. (2013)
Heat-labile (*lt*)	LT-F CACACGGAGCTCCTCAGT C LT-R CCC CCA GCC TAG CTT AGT TT	508	57°C	Talukdar et al. (2013)
Heat-stable (*st*)	ST-F GCTAAACCAGTAG AGGTCTTCAAAA ST-R CCCGGTACAGAGCAGGATTACAACA	147	57°C	Nguyen et al. (2005)
Shiga toxin (*stx1*)	stx1F-CACAATCAGGCGTCGCCAGCGCACTTGCT stx1R-TGTTGCAGGGATCAGTGGTACGGGGATGC	606	57°C	Islam et al. (2007)
Shiga toxin (*stx2*)	stx2F-CCACATCGGTGTCTGTTATTAACCACACC stx2R-GCAGAACTGCTCTGGATGCATCTCTGGTC	372	57°C	Heuvelink et al. (1995)
Invasion associated locus (*ial*)	ial upper-CTGGATGGTATGGTGAGG ial lower-GGAGGCCAACAATTATTTCC	320	57°C	Frankel et al. (1989)
Invasion plasmid antigen H (*ipaH*)	Shig-1-TGGAAAAACTCAGTGCCTCT Shig-2-CCAGTCCGTAAATTCATTCT	424	57°C	Lüscher and Altwegg (1994)
Beta-lactamase (*bla*_TEM_)	F-CATTTCCGTGTCGCCCTTATTC R-CGTTCATCCATAGTTGCCTGAC	800	58°C	Dallenne et al. (2010)
Beta-lactamase (*bla*_SHV_)	F-AGCCGCTTGAGCAAATTAAAC R-ATCCCGCAGATAAATCACCAC	713	58°C	Dallenne et al. (2010)
Beta-lactamase (*bla*_CTX−M−15_)	F-CACACGTGGAATTTAGGGACT R-GCCGTCTAAGGCGATAAACA	996	56°C	Sidjabat et al. (2009)
Beta-lactamase (*bla*_CTX−M−2_)	F-ATGATGACTCAGAGCATTCG R-TGGGTTACGATTTTCGCCGC	866	56°C	Islam et al. (2012)
Beta-lactamase (*bla*_CTX−M−1_)	F-TTAGGAARTGTGCCGCTGYA R-CGATATCGTTGGTGGTRCCAT	561	60°C	Dallenne et al. (2010)
Beta-lactamase (*bla*_CTX−M−8_)	F- AACRCRCAGACGCTCTAC R- TCGAGCCGGAASGTGTYAT	688	60°C	Islam et al. (2012)
Beta-lactamase (*bla*_OXA−1_)	F-GGCACCAGATTCAACTTCAAG R-GACCCCAAGTTTCCTGTAAGTG	564	58°C	Dallenne et al. (2010
Beta-lactamase (*bla*_CTX−M−9_)	F- ATGGTGACAAAGAGAGTGCA R- CCCTTCGGCGATGATTCTC	870	60°C	Islam et al. (2012)
Beta-lactamase (*bla*_OXA−47_)	F-TCAACTTTCAAGATCGCA R-GTGTGTTTAGAATGGTGA	609	50°C	Islam et al. (2012)
Beta-lactamase (*bla*_CMY−2_)	F-GACAGCCTCTTTCTCCACA R-TGGAACGAAGGCTACGTA	1143	50°C	Islam et al. (2012)
Beta-lactamase (*bla*_NDM−1_)	F-GGTTTGGCGATCTGGTTTTC R-CGGAATGGCTCATCACGATC	465	57°C	Islam et al. (2012)
Tetracycline (*tet*A)	F-GTAATTCTGAGCACTGTCGC R-CTGCCTGGACAACATTGCTT	937	62°C	Saenz et al. (2004)
Tetracycline (*tet*M)	F-GTGGACAAAGGTACAACGAG R-CGGTAAAGTTCGTCACACAC	406	58°C	Ng et al. (2001)
Tetracycline (*tet*B)	F-CTCAGTATTCCAAGCCTTTG R-CTAAGCACTTGTCTCCTGTT	416	57°C	Saenz et al. (2004)
Sulfonamide (*sul*1)	F-TGGTGACGGTGTTCGGCATTC R-GCGAGGGTTTCCGAGAAGGTG	789	63°C	Saenz et al. (2004)
Sulfonamide (*sul*3)	F-CATTCTAGAAAACAGTCGTAGTTCG R-CATCTGCAGCTAACCTAGGGCTTTGGA	990	51°C	Saenz et al. (2004)
Sulfonamide (*sul*2)	F-CGGCATCGTCAACATAACC R-GTGTGCGGATGAAGTCAG	722	50°C	Saenz et al. (2004)
Aminoglycoside (*ant(4´)-Ia*)	F-CTGCTAAATCGGTAGAAGC R-CAGACCAATCAACATGGCACC	172	58°C	Schmitz et al. (1999)
Quinolones (*qnr*A)	F- AGAGGATTTCTCACGCCAGG R- TGCCAGGCACAGATCTTGAC	580	56°C	Islam et al. (2012)
Aminoglycoside (*aacC*(3)-1)	F-ACCTACTCCCAACATCAGCC R-ATATAGATCTCACTACGCGC	169	60°C	Vliegenthart et al. (1991)
Quinolones (*qnr*B)	F-GGMATHGAAATTCGCCACTG R-TTTGCYGYYCGCCAGTCGAA	264	56°C	Islam et al. (2012)
Quinolones (*qnr*S)	F-GCAAGTTCATTGAACAGGCT R-TCTAAACCGTCGAGTTCGGCG	428	60°C	Farajzadeh-Sheikh et al. (2019)
Quinolones (*qnrC*)	F- GGGTTGTACATTTATTGAATC R- TCCACTTTACGAGGTTCT	307	52°C	Wang et al. (2009)
Class II integron (*int*I2)	F-CACGGATATGCGACAAAAAGG R-TGTAGCAAACGAGTGACGAAATG	788	60°C	Rizk and El-Mahdy (2017)
Class I integron (*int*I1)	F-GGTCAAGGATCTGGATTTCG R-ACATGCGTGTAAATCATCGTC	436	60°C	Rizk and El-Mahdy (2017)

### Extended-spectrum beta-lactamase detection of *E. coli*

ESBL-producing *E. coli* was detected using CHROMagar ESBL media (75006 Paris, France), following established protocols from previous studies. After an incubation period of 18–24 h at 37°C, ESBL production was identified by examining the distinct colony morphology and growth on the culture media. Colonies showing a dark pink to reddish color on CHROMagar ESBL plates indicated the likely presence of ESBL-producing *E. coli*. Colonies from positive samples underwent ESBL phenotypic confirmation through the double-disk test method, following Clinical and Laboratory Standards Institute (CLSI) guidelines (2020). This method involved standard disk diffusion with ceftazidime-clavulanate, ceftazidime, cefotaxime-clavulanate, and cefotaxime disks. After 18 h of incubation at 37°C, ESBL production was assessed based on CLSI standards (CLSI 2020), which required observing a ≥5-mm increase in the inhibition zone diameter when clavulanate was added compared to the agent test conducted alone. *Escherichia coli* ATCC 25922 was used as a control strain.

### Antimicrobial susceptibility testing profile of non-ESBL and ESBL *E. coli*

Antimicrobial susceptibility testing (AST) assessment for both ESBL *E. coli* and non-ESBL *E. coli* was assayed using a broth dilution assay, following CLSI guidelines (CLSI 2020). A total of 17 antimicrobial agents were assayed against non-ESBL and ESBL *E. coli* isolates using antimicrobials from Mast Diagnostics, UK. These included various classes such as penicillins (ampicillin 4–64 μg/ml, piperacillin 8–256 μg/ml), β-lactam combination agents (amoxicillin-clavulanate 4/2–64/32 μg/ml, ampicillin-sulbactam 8/2–256/8 μg/ml), monobactams (aztreonam 2–32 μg/ml), tetracyclines (tetracycline 2–32 μg/ml), carbapenems (imipenem 0.5–8 μg/ml), fluoroquinolones (ciprofloxacin 0.125–2 μg/ml), macrolides (azithromycin 8–64 μg/ml), nitrofurans (nitrofurantoin 16–256 μg/ml) and phenicols (chloramphenicol 4–64 μg/ml), aminoglycosides (gentamicin 2–32 μg/ml, amikacin 8–128 μg/ml), folate pathway antagonists (trimethoprim-sulfamethoxazole 1/17–8/152 μg/ml), fosfomycins (fosfomycin 32–512 μg/ml), cephems (cefotaxime 0.5–8 μg/ml, ceftazidime 2–32 μg/ml). *Escherichia coli* ATCC 25922 served as a control strain.

### Multiple AMR characterizations of the non-ESBL and ESBL *E. coli*

Non-ESBL and ESBL *E. coli* strains manifesting resistance to a minimum of three antimicrobial classes were categorized as MDR. The multiple antimicrobial resistance index (MARI) of these *E. coli* isolates was assessed using the formula:


\begin{eqnarray*}
{\mathrm{MAR\ index}} = \frac{b}{c}
\end{eqnarray*}

Here, “*b*” represents the cumulative count of antimicrobials to which *E. coli* exhibited resistance. At the same time, “*c*” indicates the number of antimicrobials utilized against the bacterial species in total. An MARI exceeding 0.2 signifies intensive antimicrobial usage in the area, suggesting a high-risk environment for the proliferation of AMR.

### Extracellular virulence factors formation

Initially, *E. coli* cultures were grown overnight in TSB at 37°C. After incubation, bacterial cells were harvested via centrifugation and washed with a phosphate-buffered solution (PBS). The resulting bacterial pellet was then treated with lysozyme to degrade the peptidoglycan layer of the bacterial cell wall enzymatically. Following lysozyme treatment, the suspension underwent centrifugation to separate cell wall debris from the supernatant, which contained the released S-layer proteins. Further purification of the S-layer proteins was achieved using ultracentrifugation. The purity and concentration of the isolated proteins were assessed through sodium dodecyl sulfate polyacrylamide gel electrophoresis, followed by spectrophotometric methods for quantification. The bacterial pellet was resuspended in PBS (pH 7.2) and mixed with egg yolk phospholipid, then left to incubate at 37°C for 24 h to evaluate lecithinase activity. Afterward, the presence of lecithinase activity was determined by visually examining agar plates containing lecithin for the development of a clear zone around bacterial colonies, indicating hydrolysis of the substrate. In the DNase assay, the washed bacterial pellet was resuspended in PBS supplemented with magnesium ions (Mg^2+^). Subsequently, the bacterial suspension was incubated with purified DNA from the *E. coli* isolate. After the incubation period, the reaction mixture was analyzed to evaluate the extent of DNA degradation by the DNase enzyme produced by *E. coli*, typically through agarose gel electrophoresis to visualize DNA fragmentation. For the gelatinase activity assay, gelatin plates were prepared by incorporating gelatin into Tryptic Soy Agar (TSA) at a concentration of ∼3%–5%. After sterilization, the medium was cooled to 45°C–50°C and poured into Petri dishes, then left to solidify at room temperature. Bacterial cells were streaked onto the gelatin plate surface using a sterile loop and incubated at 37°C for 24 h. Clear zones around bacterial colonies were observed post-incubation, indicating the enzymatic breakdown of the gelatin substrate by *E. coli* gelatinase.

The hemolytic activity against red blood cells was assessed by cultivating isolates on blood agar with 10% sheep blood and incubating them for 24 h at 37°C. Positive hemolysis was confirmed by observing clear zones and a pale-yellow discoloration of the blood agar. Protease activity was evaluated by examining extracellular protease activity on TSA plates with 1% casein. Isolates were inoculated onto these plates and incubated at 37°C for 48 h. Positive results were indicated by clearance zones around colonies, indicating casein hydrolysis. Culturing isolates on TSA plates determined lipase activity with 1% Tween 80, and lipase-producing organisms were identified by a transparent halo around their growth zones after 48 h at 37°C. Curli and cellulose formation were investigated using TSA plates supplemented with Congo red and brilliant blue, with isolates cultured and incubated for 48 h at 28°C. Morphotypes reflecting various degrees of cellulose and curli formation were categorized based on observed characteristics such as roughness, dryness, and color, allowing for the classification of isolates into three distinct groups (Beshiru et al. 2022).

### The biofilm formation profile of *E. coli* isolates

Biofilm formation was executed following the procedure described previously (Igbinosa et al. 2022). Pure *E. coli* colonies were cultured in 4.5 ml of TSB and incubated for 18 h at 37°C. After centrifugation, the cell pellets were washed, suspended in PBS, and adjusted to pH 7.2. These suspensions were then inoculated into wells of 96-well microtiter plates containing cell suspensions and TSB to assess *E. coli* adherence to a solid surface, following established procedures. Negative control wells contained only TSB, while *E. coli* ATCC 25922 was the positive control. Each assay was performed in triplicate, and biofilm formation was categorized based on previously defined criteria (Igbinosa et al. 2022).

### Bacterial adherence to hydrocarbons assay

The bacterial adherence to hydrocarbons (BATH) assay, as outlined by Zoueki et al. (2010), was conducted with modifications. Instead of measuring absorbance, the initial bacterial concentration was determined using a Helber counting chamber under phase contrast microscopy. The bacterial suspension’s stock concentration was adjusted to 5 × 10^7^ cells/ml after assessment. The standard BATH procedure was followed after that, involving hydrocarbon addition, vortexing, settling, and aqueous phase extraction. A Pasteur pipette was employed to transfer a 10 μl sample onto the counting chamber, facilitating accurate enumeration using grid lines. The use of phase contrast microscopy allowed for clear differentiation between hydrocarbon droplets and bacterial cells, ensuring precise counting. This method’s precision was validated by repeated counts, which consistently showed <5% variation between measurements. The partition of cells to the hydrocarbon phase was calculated using the formula FP_N_ = 1 − *C*_f_/*C*_o_, with *C*_o_ representing the initial cell concentration and *C*_f_ representing the concentration after vortexing and phase separation.

### Salting aggregation test

The TSB cultures, incubated overnight, underwent harvesting, followed by two washes and resuspension in PBS (pH 7.2). Isolates were mixed with various concentrations of ammonium sulphate [(NH_4_)_2_SO_4_], ranging from 0.2 to 4 M, on microscope slides to stimulate aggregation. Each combination consisted of 25 μl volumes containing 2 × 10^9^ bacteria (Sorongon et al. 1991). Improved visualization of aggregation was attained by introducing 400 μl of methylene blue solution to 10 ml of (NH_4_)_2_SO_4_ solution. Slides were shaken for 2 and 4 min on a rocking shaker at room temperature, then examined visually against a white background to determine the salting aggregation test (SAT) value, representing the lowest (NH_4_)_2_SO_4_ concentration causing aggregation. Each experiment was repeated three times on two separate occasions, with matching (NH_4_)_2_SO_4_ concentrations serving as controls. The classification was based on the following criteria: <0.1 M = highly hydrophobic, 0.1–1.0 M = moderately hydrophobic, and >1.0 M = hydrophilic.

### Diarrheagenic and AMR gene profiling

The methodology involved using bacterial lysates as the DNA source for PCR to identify AMR genes and genetic elements associated with virulence in different pathotypes of DEC. Specific primers targeting genes such as bundle-forming pilus (*bfp*), heat-labile (*lt*), aggR-activated island (*aaiC*), anti-aggregation protein transporter (*aat*), attaching and effacing (*eae*), and heat-stable (*st*) were employed for PCR amplification following established protocols (Oswald et al. 2000, Nguyen et al. 2005, Mohamed et al. 2009, Talukdar et al. 2013). The PCR was conducted with cycling conditions, including an annealing temperature of 57°C for 20 s. Separate PCRs were performed for Shiga toxin genes (*stx1* and *stx2*) and invasion-associated locus (*ial*) and invasion plasmid antigen H (*ipaH*) using previously described methods (Frankel et al. 1989, Lüscher and Altwegg 1994, Heuvelink et al. 1995, Islam et al. 2007, Talukdar et al. 2013). Resistance determinants for tetracycline [*tetB, tetA, tetM*], sulfonamides [*sul3, sul2, sul1*], aminoglycosides [*aacC(3)-1, ant(4′)-Ia*], quinolones [*qnrB, qnrA, qnrS, qnrC*], and beta-lactamase [*bla*_TEM_, *bla*_SHV_, *bla*_CTX−M−15_, *bla*_CTX−M−2_, *bla*_CTX−M−1_, *bla*_CTX−M−8_, *bla*_OXA−1_, *bla*_CTX−M−9_, *bla*_OXA−47_, *bla*_CMY−2_, *bla*_NDM−1_] were assessed, as reported by Sáenz et al. (2004). The investigation also included the determination of *intI2* and *intI1* genes, which encode the class 1 and class 2 integrases, following the methodology previously outlined by Sáenz et al. (2004). Thermocycling conditions and specific primers are detailed in Table 1.

### Statistical analysis

Data analysis was conducted using Microsoft Excel and IBM SPSS. Descriptive statistics, including mean and standard deviations, were used to summarize the data. A one-way ANOVA was employed to compare multiple variables, followed by Duncan’s multiple range test. Statistical significance was considered for *P*-values <.05.

## Results

### The density of *E. coli* in the seafood samples

*Escherichia coli* population density ranged from undetectable to 5.25 log_10_ CFU/g in the samples, with a mean population count of 2.04 ± 1.28 log_10_ CFU/g (Table 2). Among the seafood sample categories, fish sauce exhibited the highest mean population density (3.95 ± 1.02 log_10_ CFU/g), followed by smoked fish (2.46 ± 1.69 log_10_ CFU/g), smoked prawns and shrimps (2.20 ± 1.36 log_10_ CFU/g), dried fish (1.15 ± 0.47 log_10_ CFU/g), crayfish (1.26 ± 0.21 log_10_ CFU/g), seafood soups and stews (1.95 ± 0.42 log_10_ CFU/g), and seafood snacks (1.85 ± 0.96 log_10_ CFU/g). A significant difference exists amongst the RTE seafood (*P *< .05). No counts were detected in canned seafood samples (Table 2).

**Table 2.** Population density of *E. coli* in the seafood samples.

RTE seafood types	Samples assessed	Range (Log10 CFU/g)	Mean counts
Smoked fish	60	Nil–4.79	2.46 ± 1.69^b^
Dried fish	60	Nil–3.45	1.15 ± 0.47^c^
Fish sauce	60	Nil–5.25	3.95 ± 1.02^a^
Crayfish	60	Nil–2.56	1.26 ± 0.21^c^
Dried prawns and shrimps	60	Nil–2.34	1.04 ± 0.65^c^
Smoked prawns and shrimps	60	Nil–4.51	2.20 ± 1.36^b^
Seafood soups and stews	50	Nil–2.26	1.95 ± 0.42^bc^
Seafood snacks	60	Nil–2.15	1.85 ± 0.96^bc^
Canned seafood	50	Nil	Nil
Total	520		

Legend: Values in log_10 _± standard deviation. Mean population density with different alphabets showed significant differences.

### Prevalence of *E. coli* in the seafood samples

Our analysis of 520 processed RTE seafood samples revealed a concerning presence of *E. coli*, with 12.1% (*n *= 63) of samples testing positive (Table 3 ). The distribution of *E. coli* contamination varied markedly across different seafood types. Smoked fish emerged as the most problematic category, with 26.7% of samples contaminated. Seafood soups and stews showed the second-highest contamination rate (22%). Smoked prawns and shrimps (15%) and fish sauce (13.3%) also showed notable contamination levels. A lower prevalence was observed in dried products like dried fish (11.7%) and dried prawns and shrimps (6.7%). Crayfish showed the lowest contamination rate (5%). The presence of ESBL-producing *E. coli* in 2.3% of all samples is particularly alarming (Table 4). The fact that ESBL-producing strains were found across various product types, including 5% of smoked fish samples, underscores the widespread nature of this threat.

**Table 3.** Prevalence of *E. coli* in the seafood samples.

RTE seafood types	Samples assessed	*Escherichia coli* positive samples
Smoked fish	60	16(26.7)
Dried fish	60	7(11.7)
Fish sauce	60	8(13.3)
Crayfish	60	3(5)
Dried prawns and shrimps	60	4(6.7)
Smoked prawns and shrimps	60	9(15)
Seafood soups and stews	50	11(22)
Seafood snacks	60	5(8.3)
Canned seafood	50	0
Total	520	63(12.1)

**Table 4.** Distribution of ESBL and non-ESBL *E. coli* in the seafood samples.

RTE seafood types	Samples assessed	Non-ESBL *E. coli*	ESBL-*E. coli*
Smoked fish	60	13(21.7)	3(5)
Dried fish	60	6(10)	1(1.7)
Fish sauce	60	5(8.3)	3(5)
Crayfish	60	3(5)	0
Dried prawns and shrimps	60	4(6.7)	0
Smoked prawns and shrimps	60	7(11.7)	2(3.3)
Seafood soups and stews	50	9(18)	2(3.3)
Seafood snacks	60	4(6.7)	1(1.7)
Canned seafood	50	0	0
Total	520	51(9.8)	12(2.3)

### Antimicrobial susceptibility profile of the *E. coli* isolates

The antimicrobial susceptibility profiles of *E. coli* isolates from seafood samples reveal a concerning prevalence of resistance, with notable differences between non-ESBL and ESBL-producing strains (Table 5). Among non-ESBL-producing *E. coli*, resistance to ampicillin was alarmingly high (76.5%), gentamicin resistance (62.7%) is particularly worrying, and high resistance rates to kanamycin (52.9%) and chloramphenicol (52.9%) and ciprofloxacin resistance (47.1%) are concerning. ESBL-producing *E. coli* isolates exhibited even more extensive resistance: 100% resistance to all tested beta-lactams and aztreonam; high resistance rates to other antibiotic classes (58.3%–66.7% for aminoglycosides, fluoroquinolones, and tetracyclines). The MARI ranged from 0 to 0.71, with 82.5% of isolates classified as MDR. This high prevalence of MDR strains (MARI > 2) indicates that these *E. coli* isolates are resistant to antibiotics from multiple classes. The fact that 87.3% of isolates were resistant to at least one antimicrobial agent highlights the pervasiveness of antibiotic resistance in this population of *E. coli* from seafood.

**Table 5.** Antimicrobial susceptibility profile of the *E. coli* isolates.

		Non-ESBL *E. coli* (*n *= 51)			ESBL-*E. coli* (*n *= 12)		
Antimicrobial class	Antimicrobial agents	R(%)	I(%)	S(%)	R(%)	I(%)	S(%)
Penicillins	Ampicillin	39(76.5)	7(13.7)	5(9.8)	12(100)	0	0
	Piperacillin	26(50.9)	11(21.6)	15(29.4)	12(100)	0	0
B-Lactam combination agents	Amoxicillin-clavulanate	13(25.5)	15(29.4)	23(45.1)	12(100)	0	0
	Ampicillin-sulbactam	8(15.7)	6(11.8)	37(72.5)	12(100)	0	0
Cephems	Ceftazidime	4(7.8)	7(13.7)	40(78.4)	12(100)	0	0
	Cefotaxime	4(7.8)	6(11.8)	41(80.4)	12(100)	0	0
Monobactams	Aztreonam	21(41.2)	12(23.5)	18(35.3)	12(100)	0	0
Carbapenems	Imipenem	0	0	51(100)	0	0	12(100)
Aminoglycosides	Gentamicin	32(62.7)	9(17.6)	10(19.6)	8(66.7)	3(25)	1(8.3)
	Kanamycin	27(52.9)	12(23.5)	12(23.5)	7(58.3)	4(33.3)	1(8.3)
Macrolides	Azithromycin	11(21.6)	NA	40(78.4)	3(25)	NA	9(75)
Tetracyclines	Tetracycline	22(43.1)	9(17.6)	20(39.2)	8(66.7)	2(16.7)	2(16.7)
Fluoroquinolone	Ciprofloxacin	24(47.1)	11(21.6)	16(31.4)	7(58.3)	1(8.3)	4(33.3)
Folate pathway antagonists	Trimethoprim-sulfamethoxazole	22(43.1)	8(15.7)	21(41.2)	7(58.3)	2(16.7)	3(25)
Phenicols	Chloramphenicol	27(52.9)	8(15.7)	16(31.4)	6(50)	2(16.7)	4(33.3)
Fosfomycin	Fosfomycin	14(27.5)	11(21.6)	26(50.9)	5(41.7)	4(33.3)	3(25)
Nitrofurans	Nitrofurantoin	2(3.9)	12(23.5)	37(72.6)	6(50)	3(25)	3(25)

Legend: AMP: ampicillin, PRL: piperacillin, AML: amoxicillin-clavulanate, SAM: ampicillin-sulbactam, TAZ: ceftazidime, CTX: cefotaxime, AZT: aztreonam, IPM: imipenem, GEN: gentamicin, HLK: kanamycin, AZM: azithromycin, TET: tetracycline, CIP: ciprofloxacin, SXT: trimethoprim-sulfamethoxazole, CHL: chloramphenicol, FOS: fosfomycin, NIT: nitrofurantoin, NA: not applicable.

### Assessment of extracellular virulence factors and biofilm formation in *E. coli* isolates from the RTE seafoods

**Figure 1.**
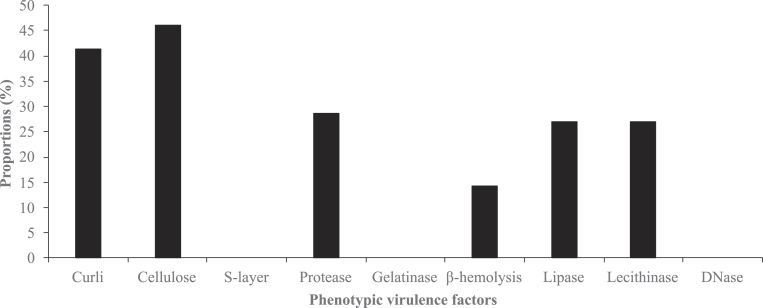
Phenotypic virulence factor expression in *E. coli* isolates.

**Figure 2.**
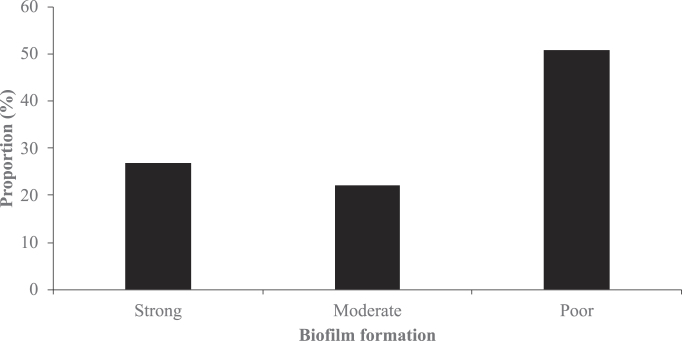
Biofilm formation of *E. coli* isolates.

The *E. coli* isolates exhibited a diverse array of extracellular virulence factors, with cellulose 46.0% (*n = *29) and curli 41.3% (*n = *26) being the most prevalent (Fig. 1 ). These two factors are known to play crucial roles in biofilm formation, which is reflected in the significant positive correlations observed between biofilm formation and both cellulose (*r *= 0.446, *P *< .01) and curli (*r *= 0.615, *P *< .01). Interestingly, while about half of the isolates, 50.8% (*n = *32), showed poor biofilm formation capabilities, a substantial proportion demonstrated either strong 26.9% (*n = *17) or moderate 22.2% (*n = *14) biofilm-forming ability (Fig. 2). The strong correlations between biofilm formation and surface hydrophobicity measures (SAT: *r *= 0.914, *P *< .01; BATH: *r *= 0.922, *P *< .01) further underscore the importance of cell surface properties in facilitating bacterial adhesion and subsequent biofilm development. Moreover, the significant associations between biofilm formation and various virulence factors such as lipase (*r *= 0.465, *P *< .01), protease (*r *= 0.301, *P *< .05), and specific virulence genes (*aai, lt, ipaH*) indicate that biofilm-forming ability may be linked to overall pathogenicity. The presence of β-hemolysis in 14.3% (*n = *9) of isolates is noteworthy due to its role in tissue damage and nutrient acquisition during infection.

### Surface hydrophobicity of *E. coli* isolates from RTE seafood

**Figure 3.**
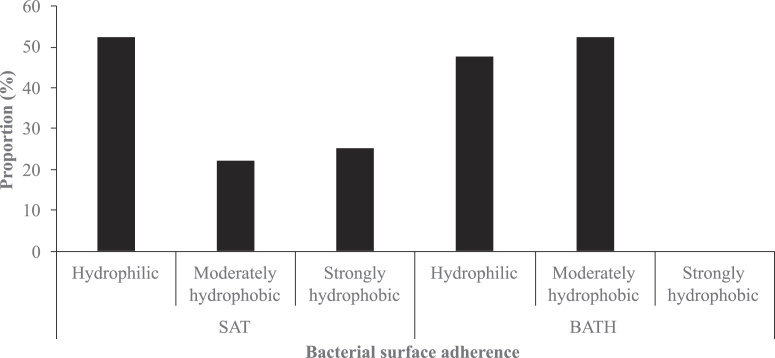
Surface adherence patterns of *E. coli* isolates.

The hydrophobicity assays revealed a diverse range of surface properties among the *E. coli* isolates, with important implications for their behavior in various environments. Using the BATH method, we observed a nearly even split between hydrophilic 47.5% (*n = *30) and moderately hydrophobic 52.4% (*n = *33) isolates (Fig. 3). The SAT provided a more nuanced view of hydrophobicity, revealing three distinct groups: hydrophilic 52.4% (*n = *33), moderately hydrophobic 22.2% (*n = *14), and strongly hydrophobic 25.4% (*n = *16). Interestingly, the two methods yielded slightly different proportions of hydrophilic isolates (47.5% in BATH vs. 52.4% in SAT), highlighting the importance of using multiple techniques to assess bacterial surface properties. The heterogeneity in hydrophobicity profiles observed across the *E. coli* population suggests adaptability to diverse ecological niches.

### Distribution of diarrheagenic factors among the seafood samples positive for *E. coli*

The analysis of *E. coli*-positive seafood samples revealed a concerning presence of various diarrheagenic determinants (Table 6 ). The most prevalent virulence genes were *aai* and *eae*, both detected in 15.9% (*n = *10) of the samples. Interestingly, while the *aai* gene was frequently detected, the full complement of genes necessary for typical EAEC (tEAEC) was only found in 6.3% (*n = *4) of samples. Indeed, atypical EAEC (aEAEC) was the most common atypical pathotype, occurring in 15.9% (*n = *10) of samples (Table 6). The presence of Shiga toxin-producing genes (*stx1* and *stx2*) in 3.2% (*n = *2) of samples, although relatively low, is particularly concerning. Notably, the study revealed a higher prevalence of atypical pathotypes compared to their typical counterparts across all categories (EAEC, EPEC, ETEC, STEC, and EIEC) (Table 7).

**Table 6.** Distribution of diarrheagenic determinants among the *E. coli*-positive seafood samples.

		Diarrheagenic determinants									
RTE seafood types	*Escherichia coli* positive samples	*aai*	*aat*	*eae*	*bfp*	*lt*	*st*	*stx1*	*stx2*	*ial*	*ipaH*
Smoked fish	16(26.7)	4(25)	2(12.5)	4(25)	2(12.5)	2(12.5)	2(12.5)	1(6.25)	–	1(6.25)	–
Dried fish	7(11.7)	–	1(14.3)	1(14.3)	–	1(14.3)	–	–	–	–	1(14.3)
Fish sauce	8(13.3)	2(25)	1(12.5)	–	1(12.5)	1(12.5)	1(12.5)	–	1(12.5)	1(12.5)	1(12.5)
Crayfish	3(5)	–	–	–	–	1(33.3)	1(33.3)	1(33.3)	–	–	–
Dried prawns and shrimps	4(6.7)	–	1(25)	1(25)	1(25)	–	–	–	–	–	1(25)
Smoked prawns and shrimps	9(15)	2(22.2)	1(11.1)	–	–	2(22.2)	1(11.1)	–	1(11.1)	–	–
Seafood soups and stews	11(22)	1(9.1)	2(18.2)	3(27.3)	3(27.3)	1(9.1)	–	–	–	1(9.1)	2(18.2)
Seafood snacks	5(8.3)	1(20)	–	1(20)	1(20)	1(20)	–	–	–	–	–
Canned seafood	0	–	–	–	–	–	–	–	–	–	–
Total	63(12.1)	10(15.9)	8(12.7)	10(15.9)	8(12.7)	9(14.3)	5(7.9)	2(3.2)	2(3.2)	3(4.8)	5(7.9)

**Table 7.** Distribution of typical diarrheagenic *E. coli* in the seafood samples.

		Diarrheagenic determinants				
		EAEC	EPEC	ETEC	STEC	EIEC
RTE seafood types	*Escherichia coli* positive samples	*aai + aat*	*eae + bfp*	*lt + st*	*stx1 + stx2*	*ial + ipaH*
Smoked fish	16(26.7)	1(6.3)	2(12.5)	1(6.3)		
Dried fish	7(11.7)					
Fish sauce	8(13.3)	1(12.5)		1(12.5)		
Crayfish	3(5)			1(33.3)		
Dried prawns and shrimps	4(6.7)		1(25)			
Smoked prawns and shrimps	9(15)	1(11.1)				
Seafood soups and stews	11(22)	1(9.1)	1(9.1)			1(9.1)
Seafood snacks	5(8.3)		1(20)			
Canned seafood	0					
Total	63(12.1)	4(6.3)	5(7.9)	3(4.8)		1(1.6)

### AMR genes of *E. coli* from RTE seafood

**Figure 4.**
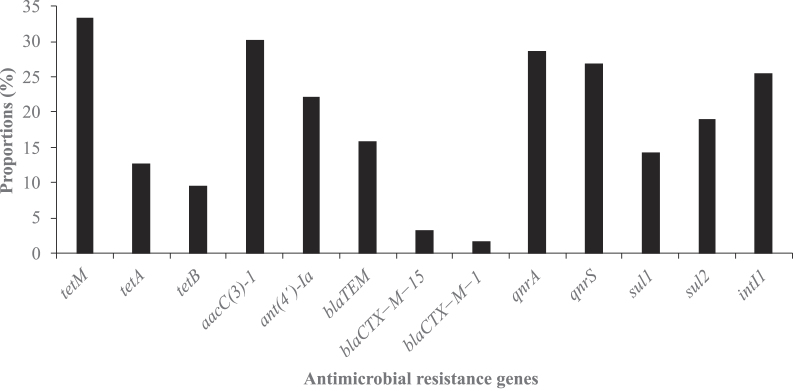
Genes associated with AMR in isolates of *E. coli*.

The analysis of ARGs in *E. coli* isolates from seafood samples revealed a concerning prevalence and diversity of resistance mechanisms, with 74.6% (*n *= 47) of isolates harboring at least one tested AMR gene (Fig. 4). Tetracycline resistance genes were particularly prevalent, with *tetM* being the most common at 33.3% (*n *= 21), followed by *tetA* at 12.7% (*n *= 11) and *tetB* at 9.5% (*n *= 6) (Fig. 4). Aminoglycoside resistance genes were also frequently detected, with *aacC(3)-1* and *ant(4′)-Ia* present in 30.2% (*n *= 19) and 22.2% (*n *= 14) of isolates, respectively. The presence of ESBL genes, although at lower frequencies (*bla_TEM_* 15.9%: *n *= 12, *bla_CTX−M_*_−15_ 3.2%: *n *= 2, and *bla_CTX−M_*_−1_ 1.6%: *n *= 1), is particularly problematic. Quinolone resistance genes (*qnrA* 28.6%: *n *= 18 and *qnrS* 26.9%: *n *= 17) were detected at alarming rates. The presence of *sul1* 14.3% (*n *= 9) and *sul2* 19% (*n *= 12) genes indicates resistance to sulfonamides, while the integrase gene *intl* 25.4% (*n *= 16) suggests the potential for horizontal gene transfer of resistance determinants. Interestingly, several AMR genes were notably absent, including *bla_SHV_, bla_CTX−M_*_−2_, *bla_CTX−M_*_−8_, *bla_OXA_*_−1_, *bla_CTX−M_*_−9_, *bla_OXA_*_−47_, *bla_CMY_*_−2_, *bla_NDM_*_−1_, *sul3, qnrB, qnrC*, and *intI2*.

## Discussion

### Comparative analysis of *E. coli* densities in RTE seafood: implications for food safety

The density of *E. coli* in RTE seafood samples is a critical indicator of contamination and food safety risks. Levels <20 CFU/g are deemed acceptable, while those between 20 and <100 CFU/g are of concern for RTE foods (Health Protection Agency 2009). In contrast to our findings, Igbinosa et al. (2020) observed *E. coli* counts ranging from 1.8 ± 0.16 to 4.1 ± 0.10 CFU/g in RTE foods, while Peter et al. (2023) reported a mean count of 6.24 log CFU/g. Aboagye et al. (2020) noted lower average counts of 2.27 log CFU/g in processed fish samples. Ramires et al. (2020) observed enumeration ranging from 21 to 27 log MPN per gram in their study. These variations highlight differences in contamination levels across seafood products and processing methods, underscoring the heterogeneous nature of *E. coli* contamination. This diversity suggests varied sources and contamination levels within the seafood supply chain, accentuating the need for vigilant monitoring and stringent food safety measures to address *E. coli* risks in RTE seafood. Analysis of *E. coli* densities across seafood types offers insights into contamination’s distribution and severity. These insights include the identification of high-risk products, processing vulnerability assessment, environmental factor correlation, efficacy of current safety measures, consumer risk stratification, regulatory focus areas, industry-specific interventions, and trend analysis over time.

### ESBL-producing *E. coli* in seafood: prevalence, risks, and mitigation strategies

The presence of ESBL-producing *E. coli* poses serious concerns due to its resistance to multiple antimicrobials, potentially leading to severe health issues if consumed. Strict food safety protocols are crucial during processing, especially for smoked fish samples, to minimize contamination risks from ESBL- and non-ESBL-producing strains. These protocols should include the implementation of Hazard Analysis and Critical Control Points systems; regular sanitation and disinfection of processing equipment and surfaces; proper personal hygiene practices for food handlers, including handwashing and use of protective clothing; temperature control throughout the processing chain, including during smoking, storage, and transportation; use of potable water for processing and cleaning; separation of raw and processed products to prevent cross-contamination, regular microbiological testing of products and processing environments; proper waste management and pest control measures; employee training on food safety practices and the importance of adhering to protocols; and maintaining detailed records for traceability and quality assurance. In comparison to our results, Shahreza (2022) reported a similar prevalence of 10.36% in Isfahan province, Iran, while Dumen et al. (2020) found a higher occurrence at 18.71% in Istanbul, Turkey. Harada et al. (2018) isolated Enterobacteriaceae species, including *E. coli*, from 75.5% of RTE fish and seafood products in Osaka Prefecture, Japan. Peter et al. (2023) reported *E. coli* isolation from 65.5% of RTE shrimp samples in Ibadan, Nigeria. Li et al. (2020) detected *E. coli* at a rate of 12.9% in RTE food in northern Xinjiang, China, while Sabala et al. (2021) recovered *E. coli* from 53% of RTE food in Mansoura city, Egypt. The studies underline diverse *E. coli* prevalence rates in seafood samples, stressing comprehensive surveillance for food safety. Urgent measures are needed to control AMR strains, ensure public health, and reduce treatment failures. Continuous monitoring and regulation are essential for significantly reducing the risk of *E. coli* contamination in seafood and minimizing associated foodborne illnesses. This risk-based approach can greatly enhance seafood safety and public health protection.

### Antimicrobial resistance profiles of *E. coli* in seafood: patterns, implications, and mitigation strategies

The antimicrobial susceptibility profile of *E. coli* isolates reveals a concerning prevalence of AMR, especially among ESBL- and non-ESBL-producing strains. A high proportion of non-ESBL-producing *E. coli* exhibited resistance to several antimicrobials. This resistance is particularly alarming among ESBL-producing *E. coli* isolates, indicating the potential ineffectiveness of conventional antimicrobial treatments and posing a significant threat to public health. Shahreza (2022) reported resistance against various antimicrobials in Isfahan province, Iran, with varying rates, while Sivaraman et al. (2020) observed high resistance to cefotaxime and cefepime in Assam, India. Studies by Ryu et al. (2012) in Seoul, Korea, Li et al. (2020) in Xinjiang, China, and others highlighted resistance patterns across different settings, showcasing the complex nature of AMR in *E. coli*. Varying degrees of resistance among ESBL-producing *E. coli* isolates underscore the need for tailored treatment strategies. Resistance to important antimicrobials like gentamicin and tetracycline further complicates the challenge of managing infections. Urgent antimicrobial stewardship efforts are necessary to promote prudent antimicrobial use alongside enhanced surveillance and implementing measures to control infection and reduce the spread of AMR *E. coli* strains, protecting public health and preserving antimicrobial effectiveness.

### Multidrug resistance in *E. coli*: prevalence, comparisons, and public health implications

Multidrug-resistance strains exhibit resistance to multiple classes of antimicrobials, posing challenges in treatment. An MARI value >2 indicates resistance to antimicrobials from at least two classes, limiting treatment options and potentially worsening infections. Our study found *E. coli* MARI ranged from 0 to 0.71, with 82.5% classified as MDR and MARI >2. In contrast, Sivaraman et al. (2020) reported an MARI of 0.26–0.63, indicating resistance to up to 12 antimicrobials. Saharan et al. (2020) found >89% resistance to >3 antimicrobials and 85.71% of ESBL-producing *E. coli* were MDR, according to Sivakumar et al. (2021). Multidrug-resistance strains were prevalent in pangasius and shrimp’ samples (Saharan et al. 2020). These findings highlight widespread MDR *E. coli* across studies, urging action to address AMR. Urgent comprehensive antimicrobial stewardship is needed to combat MDR spread. Judicious antimicrobial use is crucial in agricultural and clinical settings to curb MDR dissemination and development. The occurrence of MDR *E. coli* in seafood raises food safety concerns and public health risks. Consumption of contaminated seafood may lead to difficult-to-treat infections, contributing to global AMR burden. Continued surveillance, research, and interventions are vital to address AMR and preserve antimicrobial effectiveness.

### Virulence factors and biofilm formation in *E. coli*: pathogenic potential and public health significance

The distribution of biofilm formation and extracellular virulence factors in *E. coli* isolates showed insights into their pathogenic potential and environmental persistence. The presence of factors like curli, cellulose, protease, β-hemolysis, lipase, and lecithinase highlights diverse disease-causing mechanisms. These factors aid bacterial adhesion, immune evasion, and tissue damage, enhancing pathogenicity. High prevalence underscores the need to understand *E. coli* pathogenesis and develop mitigation strategies. Cellulose and curli contribute to biofilm formation, promoting surface attachment and protection against stresses and host defenses. Their prevalence suggests *E. coli*’s biofilm-forming potential, complicating infections and treatment. In contrast to our findings, Gupta et al. (2013) found that all isolates were hemolytic, with cytotoxicity indicators varying from 16.67% to 35.19%. At the same time, our study found that the proportions of similar factors differed, suggesting strain-specific virulence variations. Understanding these diverse virulence and biofilm formation profiles in *E. coli* isolates is crucial for assessing pathogenicity and public health implications in the following ways: risk assessment, treatment strategies, prevention measures, outbreak management, vaccine development, diagnostic improvements, public health policy, and research priorities.

### Biofilm formation and surface properties of *E. coli*: mechanisms, variability, and implications for pathogenesis

Biofilm formation enhances bacterial survival in diverse environments, posing challenges for food safety and healthcare. Gupta et al. (2013) reported weak biofilm formation in contrast to our findings, while Ramires et al. (2020) observed biofilm formation on stainless steel surfaces, suggesting strain and environmental influence. Understanding virulence and biofilm capabilities is crucial for infection prevention and improving public health outcomes. *E. coli* hydrophobicity assays, including BATH and SAT, reveal surface properties impacting interactions. Heterogeneity in hydrophobicity reflects varied ecological niches, affecting adherence to surfaces. Hydrophobic interactions drive adhesion, biofilm formation, and colonization, with factors such as curli, cellulose, protease, and lipase showing strong positive associations. Additionally, genes associated with virulence, including *aaiC* and *lt*, along with *ipaH*, displayed notable correlations with biofilm formation, with other studies reporting virulence associations with biofilm formation (Mirzahosseini et al. 2023). Furthermore, both SAT and BATH exhibited significant positive correlations with biofilm formation, highlighting the multifactorial nature of biofilm development in *E. coli* and the importance of understanding these interactions in microbial behavior and pathogenesis.

### Diarrheagenic *E. coli* in seafood: genetic diversity, prevalence, and food safety implications

The occurrence of DEC strains in seafood samples poses potential health risks. Studies show diverse patterns of diarrheagenic gene detection. Li et al. (2020) detected *stx_2_, stx_1_*, and *eae* genes, while Gupta et al. (2013) found *stx_1_* and *stx_2_* genes, with a smaller proportion positive for both. Ramires et al. (2020) identified *stx_1_, stx_2_*, and *eae* genes, whereas Lima et al. (2017) reported their absence. Typical pathotypes like tEAEC, tEPEC, tETEC, and tEIEC underscore *E. coli*’s varied pathogenic potential. Our findings and others indicate diverse DEC strains in seafood, posing risks to consumers. Sivakumar et al. (2021) identified EPEC and ETEC strains but not *bfp* or *st* genes. Similarly, Sánchez et al. (2021) found STEC strains with *stx_2_* predominant. Aboagye et al. (2020) isolated EPEC from fresh and processed fish samples, suggesting contamination risks. Ensuring food safety through stringent regulations, hygiene practices, and regular testing is crucial to mitigate *E. coli*-related illnesses from seafood consumption.

### Antimicrobial resistance genes in *E. coli*: prevalence, diversity, and comparative analysis

The findings from our study underscore a troubling prevalence of AMR genes among the identified *E. coli* isolates. Notably, key genes such as *tetM, tetA*, and *aacC(3)-1*, associated with resistance to tetracycline and aminoglycoside antibiotics, were detected, along with others like *qnrA* and *qnrS*, indicating resistance to fluoroquinolone antibiotics. The detection of these genes highlights the widespread distribution of AMR, with 74.6% of isolates harboring at least one tested AMR gene. These findings collectively emphasize the genetic basis for resistance in our *E. coli* strains. Remarkably, certain commonly associated AMR genes, such as *bla_CTX−M−2_* and *bla_SHV_*, were absent in our isolates, indicating variations in AMR profiles. Comparatively, findings from other studies reveal variations in the distribution and prevalence of AMR genes among *E. coli* isolates. For instance, Sivaraman et al. (2020) reported fewer AMR genes per isolate, typically ranging from 3 to 5 genes. Ryu et al. (2012) observed a lower prevalence of certain resistance genes like *aadA* and class 1 integron compared to our study. Li et al. (2020) identified a broader range of AMR genes in their isolates, including *tetA, tetB, bla_OXA_, bla_TEM_, floR, sul1, sul2, aadA, aadB, strA*, and *strB*, among others. Additionally, Sabala et al. (2021) and Sivakumar et al. (2021) reported the detection of unique resistance genes like *mcr-1, bla_CTX-M-28_, AmpC*, and *int1*, indicating diverse resistance mechanisms. While some similarities exist in the observed AMR gene profiles across studies, notable differences suggest variations in prevalence and distribution among strains of *E. coli* from different sources and geographic locations. These findings underscore the necessity for continuous surveillance and understanding of local AMR profiles to inform targeted interventions and control strategies against AMR in foodborne pathogens.

Our findings underscore the need for comprehensive strategies to address *E. coli* contamination and AMR in RTE seafood. We recommend future research focusing on improved hygiene protocols, antibiotic stewardship, biofilm disruption, and genetic studies of resistance mechanisms. Public health interventions should include enhanced surveillance, stricter regulations, improved processing practices, and responsible antibiotic use in aquaculture. Consumer education, international cooperation, and innovative food safety technologies are crucial. Implementing these recommendations will contribute to mitigating risks associated with *E. coli* and AMR in seafood, ultimately enhancing global food safety.

## Conclusion

Our study highlights the presence of *E. coli* in RTE seafood from Bayelsa, Nigeria, underscoring the critical need for stringent hygiene and processing standards. We found significant AMR, urging enhanced stewardship measures and regulatory oversight. Detection of virulence factors underscores the potential for gastrointestinal infections, requiring comprehensive surveillance. Our findings stress the significance of ongoing efforts to tackle microbial contamination and AMR, especially in developing nations like Nigeria. Collaboration among stakeholders is vital for public health protection and sustainable seafood production. Continued research is crucial for evidence-based interventions to maintain global seafood safety and quality.

## Data Availability

The data are made available in the manuscript. Additional data will be made available upon request.
